# Cost, outcomes, treatment pathways and challenges for diabetes care in Italy

**DOI:** 10.1186/1744-8603-10-58

**Published:** 2014-07-14

**Authors:** Federico Grimaccia, Panos Kanavos

**Affiliations:** 1 LSE Health, The London School of Economics and Political Science, Houghton Street, London WC2A 2AE, UK; 2 Dipartimento Scienze Biomediche e Sanità Pubblica, Università Politecnica delle Marche (UNIVPM), via Tronto, 10/A, Torrette di Ancona, AN 60126, Italy

**Keywords:** Diabetes, Italy, Complications, Costs, Outcomes

## Abstract

**Background:**

In Italy both incidence and prevalence of diabetes are increasing and age at diagnosis is decreasing in type 2 diabetes. Diabetes is one of the major causes of morbidity in Italy, causing several disabilities and affecting the economically active population. The objective of this paper is to identify and discuss costs, outcomes and some of the challenges of diabetes care in Italy in the context of recent policy changes.

**Methods:**

The study collected data and evidence from both primary and secondary sources. A total of 10 experts, including clinicians (diabetologists/endocrinologists) and decision makers, both at national and regional levels, were interviewed through face-to-face semi-structured interviews. The secondary sources include peer review literature from Medline, grey literature, reports from national and international sources, including professional bodies and organizations.

**Results:**

The total direct cost of diabetes for the Italian NHS in 2012 is estimated to be above €9 billion, of which more than half for hospital admissions (57%), and the remaining half for drugs (30%) and outpatient care (13%). However, there is scant evidence on indirect and intangible costs of diabetes in Italy. The quality of care addressed via the AMD Annals found overall good performance with both process and intermediate outcome indicators showing positive and improving results from 2004 to 2011, except for few parameters, including renal function and foot monitoring, which are still inadequate. Major challenges are the rising diabetes prevalence, the difficulty in meeting the rising demand for care and the scant development of multidisciplinary delivery of care, especially in the predominantly ambulatory setting of the Southern diabetes centres.

**Conclusions:**

Prevention of diabetes, especially adopting a multi-sectorial approach, should be further empowered by policy makers through promoting healthy lifestyles and specifically targeting child obesity. Other key strategies include more information and education, better diabetes management through the adoption of a chronic model of care, more focus on appropriateness and efficiency of care and better communication between diabetes centres within every Region.

## Background

In Italy both incidence and prevalence of diabetes are increasing and age at diagnosis is decreasing in type 2 diabetes. According to the national statistical source, diabetes prevalence has increased from 3.9% in 2001 to 5.4% in 2013, representing currently more than 3 million Italians with diabetes [1]. Diabetes prevalence is higher in men (5.6%) than women (5.3%) and increases with age reaching a prevalence of 20.4% in people older than 75. In general, prevalence is higher in the South of Italy and the Islands (6.6%) than in the Centre and North of Italy (respectively 5.3% and 4.6%). Furthermore, prevalence seems to be also related to education and social class, with higher rates in people with no or little education and low income [2]. Italy offers a well developed system in Europe with regard to diabetes care, being the first European Country to issue a specific law on diabetes, the Law 115 of 1987 [3]. One of the main contributions of this law is the institutionalization of diabetes centres which have since developed to form a wide network throughout Italy. There are currently approximately 680 diabetes centres evenly distributed throughout the 20 Italian Regions, roughly one centre every 100.000 inhabitants [4]. This high distribution of diabetes centres enables decentralized care, with most diabetic patients referred to their local diabetes centre. The organization of diabetes care is ensured by dedicated diabetes Commissions, both at national and regional level. The National Diabetes Commission was created by the Ministry of Health in 2003 to coordinate all stakeholders involved in diabetes care as well as monitor the implementation of the guidelines set by Law 115. The Commission includes representatives from all the main stakeholder groups dealing with diabetes: the Ministry of Health (General Direction of Planning and General Direction of Prevention), regional delegates, scientific societies, voluntary associations and patient associations [5]. Similarly, Regional Diabetes Commissions, represented by all local diabetes stakeholders, aim to coordinate activities and initiatives at regional level. Italy has just recently issued the first National Diabetes Plan in response to the European Parliament resolution of 12 March 2012 [6]. The objective of the National Plan is to provide guidelines to improve the quality of diabetes care and consistency among programmes and initiatives implemented at regional level. In Italy all diabetes care expenses are fully covered by the National Health Service (NHS), with no out-of-pocket payments. In fact, diabetes care includes the supply of all drugs and devices according to the Livelli Essenziali di Assistenza (LEA) (literally “minimum levels of care”) which represent the minimum services which must be guaranteed by all Italian Regional Health Systems [7]. This paper aims at identifying the direct costs of diabetes, presenting diabetes outcomes and quality of care and finally discussing the challenges which diabetes brings for the foreseeable future in Italy.

## Methods

The study collected data and evidence from both primary and secondary sources. A total of 10 experts, including clinicians (diabetologists/endocrinologists) and decision makers, both at national and regional levels, were interviewed face-to-face using a semi-structured questionnaire between December 2011 and October 2012. Clinicians were interviewed to address the clinical aspects of diabetes such as diagnosis and treatment. Moreover, two of them were specifically selected to give different points of view. In particular, one is a renowned diabetologist who undertakes research on direct costs of diabetes using the largest database of diabetic patients in Italy. The other clinician is the coordinator of the Annals of the Associazione Medici Diabetologi (AMD) which represents the main initiative at national level to monitor diabetes care quality in Italy. Two national decision makers from the Ministry of Health were interviewed to investigate their perspectives on the challenges posed by diabetes at national level. In addition, three regional diabetes decision makers from Lombardia, Marche and Puglia were identified to represent regional variations in organization of diabetes care between North, Centre and South. The experts were asked about the various elements and pathways of diabetes care (including prevention, diagnosis, treatment, managing of complications, follow-up and management).

Secondary data sources include peer review literature from Medline, grey literature, reports from national and international sources, including professional organizations. The following key words were used, both in English and Italian: “Italy + diabetes”; “Italy + diabetes + prevalence”; “Italy + diabetes + costs”; “Italy + diabetes + costs + complications”; “Italy + diabetes + outcomes”; “Italy + diabetes + guidelines”. Studies were selected on the basis of their abstracts or executive summaries. Articles and reports were included based on their relevance with any of the following variables: costs, treatment and quality of diabetes care. The selected articles were then read in full to extract data of interest.

## Result and discussion

### Cost of diabetes in Italy

All recent studies on cost of diabetes in Italy have been undertaken at a local level. Only one study has been conducted at national level in Italy to date. This was undertaken in 1998 as part of the wider CODE 2 (Cost of Diabetes in Europe-Type 2) study which involved 8 European Countries [8]. This study was an observational bottom-up study of 1,263 patients selected from diabetes centres (mainly located in public hospitals) and general practitioners (GPs) via questionnaires. According to the results of the study the cost per diabetic patient in Italy was circa 6 million lire (€2,991) [8], more than double (221%) the average health care expenditure per person. The total Italian NHS diabetes expenditure was approximately 10,500 billion Italian lire (≈ €5.1 billion) [8], representing 6.65% of the total health expenditure (both public and private). Overall, the direct costs accounted for 95.5% with the remainder 4.5% represented by indirect costs. The CODE 2 study also took into account the intangible costs of type 2 diabetics, showing that the quality of life was 10% lower than the general population. Although the CODE 2 data is more than a decade old, it is the only study to date looking at direct, indirect and intangible costs of diabetes in Italy. More recent analyses of direct costs of diabetes (both type 1 and 2) have been undertaken at local level using administrative data (hospital discharge files, pharmaceutical prescription and outpatient data) with a top-down approach. The ARNO-CINECA Observatory is the largest database of administrative data covering a population of approximately 10 million people living in 32 Health Local Authorities (ASL) from 8 out of the 20 Italian Regions and including almost 550,000 diabetic patients. To date, the ARNO-CINECA Observatory released two reports, the first in 2007 [9] and the second in 2011 [10] with data referring to 2006 and 2010 respectively. The ARNO-CINECA analyses compare the diabetic patients with pharmacologically treated subjects without diabetes, pair-matched for age and gender. According to the results, the average pro capita cost of a treated diabetic patient totaled €2,756 in 2010 compared to the matched non-diabetic control group of €1,545, with the extra cost being largely related to complications, primarily cardiovascular disease [10]. This represents an increase in comparison with 2006 when the same costs were respectively €2,589 and €1,682 [9]. More than half (57%) of the total diabetes costs in 2010 were for hospital admissions, while the remaining part was spent on drugs (30%, of which only 21% was for glucose-lowering drugs) and on outpatient care (13%). Based on these data, the total direct cost of diabetes for the Italian NHS in 2012 has been estimated to be above €9 billion, corresponding to almost 10% of the total NHS expenditure (unpublished observations during interview with key expert). This represents a substantial increment in comparison to the €6,64 billion estimated for 2006. A number of other smaller local studies, which also used administrative data in a top-down fashion, confirmed the high impact of complications in driving the cost of diabetes. The Emilia Romagna Region found that 18% of the total health expenditure in 2007 was due to type 2 diabetes. Resources absorbed by diabetic patients were almost threefold compared to patients without diabetes (€3,124 vs. €1,124/patient annually), mostly due to the complications such as infarction and kidney disease [11]. In 2003 the Azienda Sanitaria Locale (ASL) of Brescia undertook a similar costing analysis finding that type 1 and 2 diabetes accounted for 12% of total ASL healthcare expenditure, including hospitalization and drugs which consumed 56.8% and 27.1% respectively [12]. Costs in 2003 varied from €892 for diabetes alone to €5,330 for diabetes plus one or more co-morbidities. The number of co-morbidities increased treatment costs almost exponentially, with cardiovascular co-morbidities representing 20% of the total diabetes expenditure. Another regional study undertaken in Turin examining drug costs of type 1 and 2 diabetic and non-diabetic individuals found that annual drug costs were €830.90 per diabetic patient versus €182.80 per non-diabetic, with diabetes treatments accounting for 18.5% of total costs [13]. Overall, despite sufficient availability of cost data at local level, no study since the CODE 2 study of 1998 has been undertaken to assess national total diabetes costs. Moreover, apart from the CODE 2, no study has examined indirect diabetes costs.

### Outcomes and quality of care

**Figure 1:**
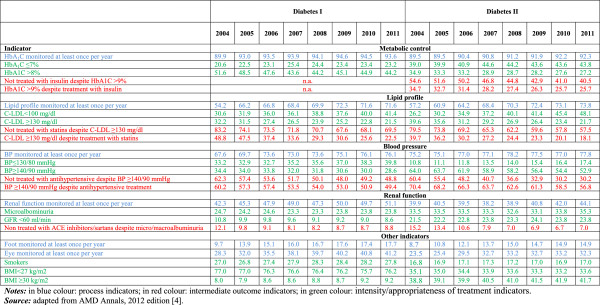
**Quality of diabetes care in the Italian diabetes centres between 2004 and 2011.** Notes: all quality indicators reported as rates (%). In blue colour: process indicators; in red colour: intermediate outcomes indicators; in green colour: intensity/appropriateness of treatment indicators.

Since 2006, the quality of care delivered by the Italian diabetes centres has been monitored by the AMD Association (Italian Association of Diabetologists). The AMD publishes on a regular basis their AMD Annals reporting a performance assessment of diabetes centres who participate voluntarily in the monitoring initiative. The number of participating diabetes centres has been steadily increasing from 180 in 2004 to the currently 320 which cover a population of almost 550,000 diabetic patients (including both patients with type 1 and type 2 diabetes) from all the 20 Italian Regions [4]. The AMD Annals include both process and intermediate outcome indicators along with few additional indicators which measure the intensity/appropriateness of treatment. Process indicators include a minimum annual monitoring of HbA_1_C levels, lipid profile, blood pressure, renal function, eye and foot conditions. Intermediate outcome indicators include the percentage of patients meeting certain parameter levels (HbA_1_C ≤7% and >8%, C-LDL <100 mg/dl and ≥130 mg/dl, blood pressure <130/80 mmHg and ≥140/90 mmHg, BMI <27 kg/m2 and ≥30 kg/m2, presence of microalbuminuria, GFR <60 ml/min) plus some other intermediate outcome indicators, such as the percentage of smokers and body weight. Intensity and appropriateness of treatment indicators measure the percentage of patients who do not reach the target levels despite the appropriate treatment as well as patients who fail to be treated despite inadequate levels of certain parameters. Final outcome indicators of diabetes-related complications currently remain outside the scope of the AMD Annals. The latest edition of the AMD Annals (2012) is a longitudinal analysis of 8 years between 2004 and 2011 [12]. The report shows an overall improvement in quality of care delivered by the participating diabetes centres both for type 1 and type 2 diabetes. Both process and intermediate outcome indicators have improved for metabolic, blood pressure and lipid profile control from 2004 to 2011 (Figure 1). The proportion of diabetic patients who had their HbA_1_C, blood pressure and lipid levels monitored increased with a subsequent raise of patients within the target levels. However, only half of the diabetic population achieved blood pressure target levels despite antihypertensive treatment, showing wide room for improvement. In addition, monitoring of renal function, eyes and foot care results still inadequate, with only a modest improvement between 2004 and 2011. Results for the intermediate outcomes pertaining to smoking and diet habits show a deterioration over time, with the number of smokers and obese patients slightly increasing in the span of 8 years.

### Challenges

Diabetes presents several challenges for both the NHS and the Italian society. The rising prevalence of diabetes represents the biggest challenge, posing a real threat in health and economic terms to the Italian health system in the future. Prevalence has been steadily increasing over the last decade, and it is projected to further increase. It has been pointed out that if prevalence continues to increase at this pace, the future impact both on society and economy will be no longer sustainable [14]. Major factors underpinning rising prevalence are the ageing population and the rising obesity. Clearly, the ageing population is difficult to control. Obesity has increased in the last years, as also shown in the AMD Annals, mostly due to the unhealthy life styles including poor diet and scarce physical activity. Obesity is so strongly related to diabetes that the two conditions together have been described as “diabesity” [14]. As a result of increasing diabetes prevalence, meeting the demand for diabetes care represents a difficult challenge, with diabetes centres struggling to provide care to a growing number of diabetic patients. Although diabetes centres are evenly distributed geographically, in highly populated Regions such as Lombardia, there is a disproportion between the low number of diabetes centres and the high number of diabetic patients. This inability to meet the demand is also evident in the rural areas, especially in the South, where the poor coordination between the professionals involved in the process of care aggravates the problem. In these areas diabetes centres have become the main reference point of care for both uncomplicated and complicated patients, instead of referring the uncomplicated patients to general practitioners (GPs) and the complicated patients to diabetes centres (unpublished observations during interview with key expert). Although prevention represents the most powerful instrument to arrest the increasing prevalence, this is scarcely appealing to politicians since the outcomes of preventive interventions take years before can be measured. Thus, from the politicians’ point of view, prevention measures are perceived as less effective than other more immediate health interventions. As a consequence, in Italy prevention is not the main focus in diabetes policy, and consequently healthcare expenditure is still more oriented towards care rather than prevention (unpublished observations during interview with key expert). An additional challenge is reflected in the organization and delivery of care between the North-Centre and the South. The Northern and Central diabetes centres are usually integrated within a hospital, providing a multi-disciplinary team including diabetologists, nurses, nutritionists and other health professionals. Moreover, the healthcare specialists treating diabetes complications (ophthalmologist, nephrologists, vascular surgeons, etc.) provide substantial and continuous support to the diabetes centres. In fact, despite they are not part of the diabetes centres’ staff, they are able to provide assistance since they work within the same facility. In contrast, the southern diabetes centres are often integrated within group practices (called “poliambulatori”) where the multi-disciplinary team is often missing leading to an often poor management of diabetes. In these settings, diabetologists generally work as independent consultants treating patients on their own without consulting with a multidisciplinary group. In addition, they undertake mainly prescribing activities (unpublished observations during interview with key expert). Another issue affecting the delivery of care is the poor compliance to diabetes treatment. This affects particularly those patients who find it difficult to follow complex treatments which require self-monitoring and self-drug administration. This problem is more relevant where a multi-disciplinary model of care is less developed, such as in the southern regions (unpublished observations during interview with key expert). Finally, although diabetes complications have been in the policy agenda of past National Prevention Plans, the current rates in Italy are still too high. Complications still represent the primary problem of diabetes, both in health and economic terms. From an economic perspective, complications account for up to half of all diabetes expenditure whereas uncomplicated diabetic patients represent a minority of NHS expenditure, as insulin and oral anti-diabetic drugs are not expensive (unpublished observations during interview with key expert). Further, also the indirect costs, mostly related to complications, account for a substantial part of the economic burden of diabetes affecting the care system, families, and the productive capabilities of diabetic patients themselves.

### Policy options

Prevention, especially through promoting healthy lifestyles, must be enforced in the policy agenda, both at national and regional levels. Primary diabetes prevention is effective in decreasing type 2 diabetes incidence and represents the key tool in arresting the raise of diabetes prevalence. In addition, prevention of complications is extremely important to decrease the impact of disability on individuals and to reduce the high costs associated with complications. Although enforcing preventive interventions will raise short term costs, it will help contain long term costs by reducing diabetes prevalence and the rate of complications. Specifically addressing obesity is a useful strategy, since obesity represents the main trigger factor for diabetes. In particular, childhood obesity should be addressed implementing nutrition courses and promoting sports and physical activity in all schools. The project “Guadagnare Salute” funded by the Ministry of Health in 2007, joining the larger “Gaining Health” project of the World Health Organization (WHO), appears to head in this direction [15]. This project promotes and coordinates a series of prevention activities across Italy addressing the four main obesity risk factors of poor diet, physical inactivity, smoking and excessive alcohol consumption. Effective prevention interventions should address also areas outside the health system which in turn impact on health. A multi-sectorial approach is more likely to be effective in achieving the desired results. For example, the cooperation of the education system will be key in preventing childhood obesity or an adequate city planning, such as supplying adequate pedestrian areas, will be crucial to boost physical activity. In this regard, Italy seems to have started employing multi-sectorial co-operation. Currently there are a number of agreements between the Ministry of Health and stakeholders outside the health sector (i.e. schools, gyms, transport, agriculture, etc.) which guarantee a common strategy towards diabetes prevention (unpublished observations during interview with key expert). In order to empower prevention, diabetes information and education are fundamental. All citizens, not only diabetic patients, must be correctly informed through awareness campaigns about the risks posed by diabetes including complications and the benefits of healthy lifestyles. In addition, diabetic patients should receive specific education on diabetes, supporting healthy lifestyles as well as treatment regimens and self-management (unpublished observations during interview with key expert). Along with prevention, another key aspect in the fight against diabetes is the adoption of a “chronic model of care” including multi-disciplinary care. Efficient cooperation between all professionals involved in diabetes care is a valuable resource to streamline care. All professionals should have their specific role so that their synergic and integrated actions can deliver the most efficient patient outcomes. Integration between GPs and diabetologists will optimize the care pathways to patients benefit. Uncomplicated patients who do not require insulin should be treated by GPs, while complicated patients who require insulin and have poor glycaemic control together with patients with a new diagnosis of diabetes should be treated by diabetologists in diabetes centres. In addition, in case of emergence of diabetes complications, diabetolgists should refer the patients to the appropriate specialist (unpublished observations during interview with key expert). In this regard, in 2006, the National Centre for the Prevention and Control of diseases (CCM, Centro Nazionale per la Prevenzione ed il Controllo delle Malattie) together with the National Health Institute (ISS, Istituto Superiore di Sanità) launched the IGEA (Integrazione, Gestione e Assistenza per la malattia diabetica) project [16]. This project provides guidelines on patient centered care disease management, and coordinates a wide range of regional activities and projects. Although some areas in Italy have already started to implement this chronic model of care, in other areas, especially in the South, this integrate approach is still largely absent. Together with a better diabetes management, appropriateness of care is another useful medium to optimize delivery of care and minimize financial resources. Recently, the Ministry of Health has been particularly concerned about appropriateness of care, commissioning a dedicated group to write guidelines on appropriateness of care, both for diabetes and obesity (unpublished observations during interview with key expert). Since the cost of diabetes and related complications are extremely high, appropriateness together with efficiency represent useful strategies to avoid the waste of unnecessary resources. Finally, improved communication and co-operation between diabetes centres is also important to optimize care delivery. Currently, Marche Region is the only region with a developed information network where all diabetes centres share the same clinical record within a unique centralized system. This enables all diabetologists to collect data and enable smooth communication between centres. Other Regions have started to follow this example, but none yet as developed as the Marche Region. Such a unique intraregional network should be empowered in all regions in order to facilitate monitoring of quality in relation to the resources employed (unpublished observations during interview with key expert).

## Conclusions

Diabetes represents a public health issue of primary importance for the Italian health system, since it is one of the major causes of morbidity. The main problem of diabetes is represented by diabetes-related complications which reduce life expectancy and cause serious disabilities such as blindness, amputation, kidney failure and heart disease. Complications represent a burden not only from a health perspective, but also from an economic perspective. Diabetes expenditure represents currently almost 10% of the total NHS expenditure, with most of the direct costs being associated with complications, primarily through hospitalization. Diabetes and related complications affects the health status of individuals and consequently their working ability and productive life. Indirect costs should therefore also be taken into account when assessing the overall economic burden of diabetes. In Italy, a better assessment of indirect and intangible costs is needed since most of the economic studies focus just on direct costs. Although quality of diabetes care seems to have improved in the last years, some parameters such as renal function, eyes examination, and foot monitoring still need to improve. Moreover, obesity and smoking have increased showing a difficulty in changing patients’ lifestyles. Overall, Italy provides a developed system of diabetes care, with numerous diabetes centres distributed throughout the country and with treatment free at point of delivery. The ageing of the Italian population and growing obesity incidence, especially in children, represents the main threats for the Italian health system. Both ageing and growing obesity incidence will drive increased diabetes prevalence in the future, impacting on population health status and affecting the NHS economy. It is essential to focus on prevention, both primary and secondary, as the main tools to reduce the incidence of type 2 diabetes-related complications. Preventive interventions may be more costly in the short term but will allow future savings. Beside prevention, an improved diabetes case management will streamline the care pathways and empower patients, as well as avoid an unnecessary resource waste. In this regard, the gap between North and South in the implementation of a “chronic model of care” should be narrowed with the South developing a better multidisciplinary and integrated approach to care.

## Abbreviations

AMD: Associazione medici diabetologi; ASL: Azienda sanitaria locale; GP: General practitioner; NHS: National Health Service.

## Competing interests

The authors declare that they have no competing interests.

## Authors’ contributions

FG made substantial contribution to the analysis and interpretation of data. Moreover, he drafted the manuscript. PK contributed in the conception and design of the study, giving final approval of the version to be published. Both authors read and approved the final manuscript.
